# Implementing IPM in crop management simultaneously improves the health of managed bees and enhances the diversity of wild pollinator communities

**DOI:** 10.1038/s41598-023-38053-5

**Published:** 2023-07-07

**Authors:** Jacob R. Pecenka, Laura L. Ingwell, Christian H. Krupke, Ian Kaplan

**Affiliations:** Department of Entomology, Purdue University, 901 W. State St., West Lafayette, IN 47907 USA

**Keywords:** Ecology, Plant sciences, Environmental sciences

## Abstract

Impacts of insecticide use on the health of wild and managed pollinators have been difficult to accurately quantify in the field. Existing designs tend to focus on single crops, even though highly mobile bees routinely forage across crop boundaries. We created fields of pollinator-dependent watermelon surrounded by corn, regionally important crops in the Midwestern US. These fields were paired at multiple sites in 2017–2020 with the only difference being pest management regimes: a standard set of conventional management (CM) practices vs. an integrated pest management (IPM) system that uses scouting and pest thresholds to determine if/when insecticides are used. Between these two systems we compared the performance (e.g., growth, survival) of managed pollinators—honey bees (*Apis mellifera*), bumble bees (*Bombus impatiens*)—along with the abundance and diversity of wild pollinators. Compared to CM fields, IPM led to higher growth and lower mortality of managed bees, while also increasing the abundance (+ 147%) and richness (+ 128%) of wild pollinator species, and lower concentrations of neonicotinoids in the hive material of both managed bees. By replicating realistic changes to pest management, this experiment provides one of the first demonstrations whereby tangible improvements to pollinator health and crop visitation result from IPM implementation in agriculture.

## Introduction

The global dependence of economically important crops on insect pollination (estimated at $215 billion globally^1^) has prompted many growers to rely on managed species, primarily the European honey bee (*Apis mellifera* L.), for supplemental pollination. However, honey bee health is increasingly threatened by agricultural intensification^2–4^. Declines in the health of honey bee colonies are well-documented^5–7^, with common drivers of colony losses identified as lack of high-quality forage^8–10^, parasites (e.g., varroa mite) and their associated diseases^11,12^, and increased toxicity of insecticides^13,14^. The effect of insecticides on honey bee colony health is especially important to consider given the dramatic increase in pesticide use on agricultural landscapes that has led to higher toxicity for invertebrates in recent decades^15–17^.

A primary contributor to insecticide hazard for bees in the US has been the rapid and widespread use of neonicotinoids, a group of systemic and highly insect-specific products that have grown into the most widely used insecticide class^18,19^. Neonicotinoids are extremely toxic to pollinators; while there is variation among active ingredients, the honey bee oral LD_50_ has been observed as low as 3.7 ng for clothianidin^20–22^. Estimates of pollen consumption throughout a honey bee worker’s life (*ca.* 100 mg^23^), mean that even brief periods of exposure in floral resources could lead to mortality of larvae or adults. With most major US row crops receiving a neonicotinoid seed treatment (NST), honey bees living within agricultural landscapes are likely exposed to these products^13,24^. Indeed, analyses of hive materials commonly report neonicotinoid residues at biologically relevant levels^25–28^. Although the combination of laboratory toxicity tests and field exposure in bee diets is often used to infer negative health outcomes, this approach has been criticized for potentially overestimating risk^29^.

Field studies examining the effect of NSTs on honey bee colonies have been conducted in different cropping systems—mostly corn (*Zea mays* L.) and canola (*Brassica napus* L.)—with mixed results. Honey bee colonies placed adjacent to fields using NSTs experienced higher worker mortality^30,31^, impaired immunity^31,32^, increased pathogen loads^32^ and reduced overwintering success^33^. However, others have found no consistent colony-level effects using similar field designs^34–37^. Even within the same study, NST-mediated impacts on honey bees can vary dramatically across landscape contexts^33^. Clearly, additional large-scale field experiments simulating realistic exposure are needed to clarify the contribution of NSTs to honey bee health in agricultural areas.

While honey bees are the most well-studied species, other managed pollinators (e.g., bumble bees (*Bombus spp.*), mason bees (*Osmia spp.*)) and native wild bees are similarly exposed to these products, potentially eliminating key contributors to crop pollination^38,39^. Non-honey bee species are important to acknowledge due to their advantages as a source of pollination: bumble bees are often more efficient pollinators^40,41^ and forage under more adverse weather conditions^42,43^ than honey bees. When multiple species were directly compared in the same experimental set-up, a few studies show that insecticides have no discernible effect on honey bees, while the same applications reduce wild bee visitation and performance^36,44^. Reproductive success and population growth of both solitary bees and social bumble bees are reduced by neonicotinoid exposure^21,33,45^ (but see Ref.^46^). Bee species respond to pesticides differently^47^ with smaller body size generally increasing vulnerability, which means that risk is greater for many of the native solitary bees^48^.

Current research approaches on how NSTs and other insecticides impact pollinators suffer from a few limitations. First, studies tend to focus on a single crop, even though many pollinators, especially generalists like honey bees and many bumble bees, forage widely across neighboring habitats and cropping systems^49–51^. Moreover, one of the most commonly field-tested systems for NST-bee interactions in the US, corn, is wind-pollinated and thus beekeepers do not intentionally place honey bees in or near these fields. Corn represents a broader land use, however, that intersects with bee forage habitat, particularly in the Midwestern US, and honey bees readily collect corn pollen in the absence of alternatives^28^. There is limited understanding of the effect that the use of corn NSTs could have on adjacent crops that require pollinators (e.g., fruits and vegetables). A second problem is that virtually all experiments employ an all-or-nothing strategy that compares the presence vs. absence of insecticides. The value of an experimental control completely free of insecticide use is debatable, depending on historical pest pressure, crop economics, and farmer behaviors. In corn^52–54^ and soybean^55–57^, for example, NSTs seem to contribute little or nothing to yield and thus an NST-free control may be appropriate. Yet, in higher-value specialty crops, foregoing insecticides altogether is unrealistic. In these systems, an insecticide-free control is theoretically useful for estimating the overall impact of insecticides on pollinators, but in practice would rarely be implemented on commercial farms. An alternative approach could employ an integrated pest management (IPM) system with economic injury levels guiding a reduced-insecticide “control” compared to a prophylactic or calendar-based spray regime. Under this scenario, the control field could be insecticide-free or have one to several applications if pest populations exceed their action threshold. Recent reviews emphasize that pollinators should be more explicitly accounted for in pest management decisions—from IPM to IPPM (integrated pest and pollinator management)^58–60^—yet we have few empirical cases documenting how IPM implementation affects pollinator health.

We conducted a multi-year, multi-site experiment across Indiana using a dual cropping system to contrast a conventional insecticide program with an IPM system, evaluating the health of both managed and wild pollinators. This experimental design placed seedless watermelon, a pollinator-dependent crop in which managed bees are deployed, within a larger corn field to simulate conditions typical for our region (Supplementary Fig. S1)^61^. Specialty crops such as watermelon are often surrounded by and rotated with row crops such as corn. We hypothesized that the reduced insecticide applications within IPM cropping system would result in healthier managed bee colonies and higher watermelon floral visitation and diversity from the wild pollinator community. Secondarily, we expected that the magnitude of response to insecticide use by managed bees would be weaker than for wild bees. This study provides some of the first data linking the adoption of IPM with a more abundant and species-rich pollinator community, a critical step to providing growers with evidence-based solutions to sustainable crop management and on-farm bee conservation.

## Results

### Honey bee colonies placed in IPM crops were more productive and heavier than in CM system

**Figure 1:**
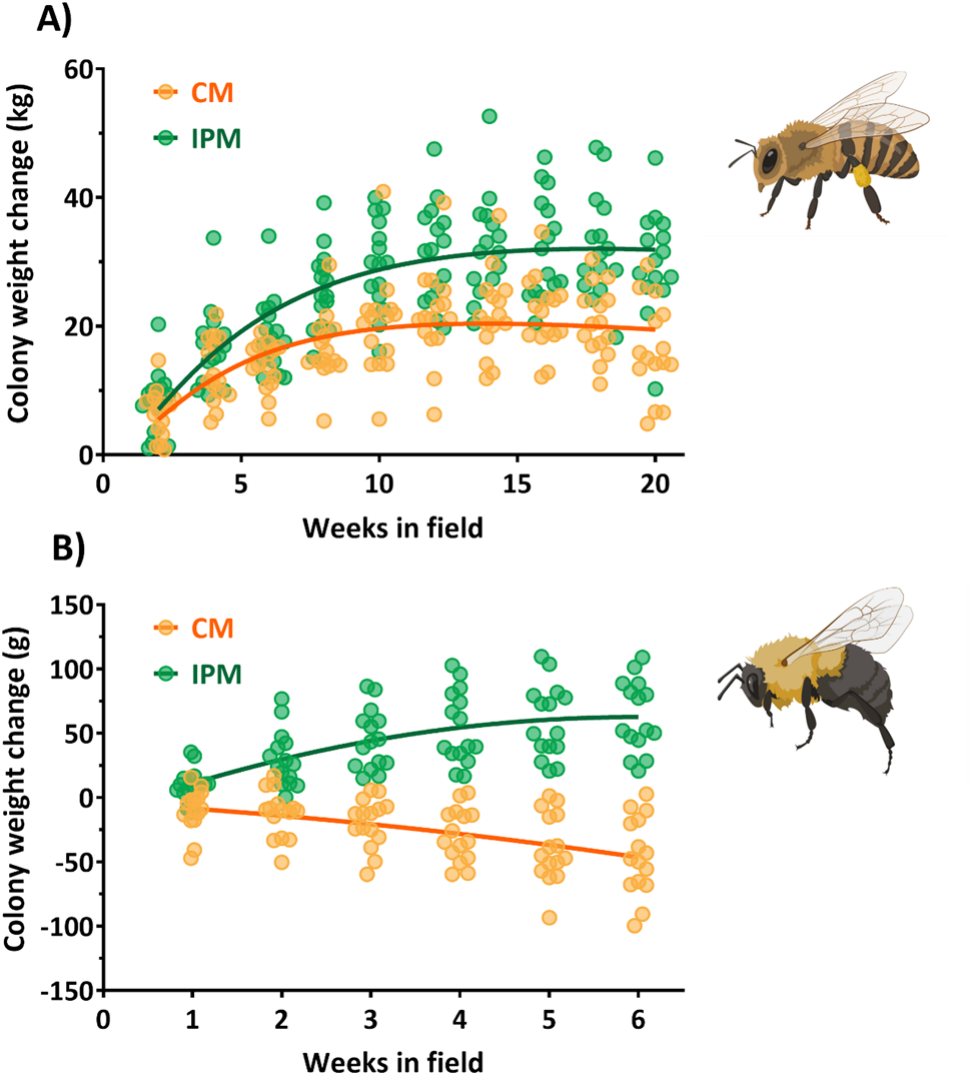
The growth of both honey bee (**A**) and bumble bee (**B**) colonies was affected by the management system of the field where they were placed. Each point is the average of all colonies—HB (n = 2) or BB (n = 4)—at each site from 2018 to 2020. Curve fit lines for both figures colonies follow a lognormal path (R^2^ for CM = 0.362 and IPM = 0.559). Bee icons from BioRender.

**Figure 2:**
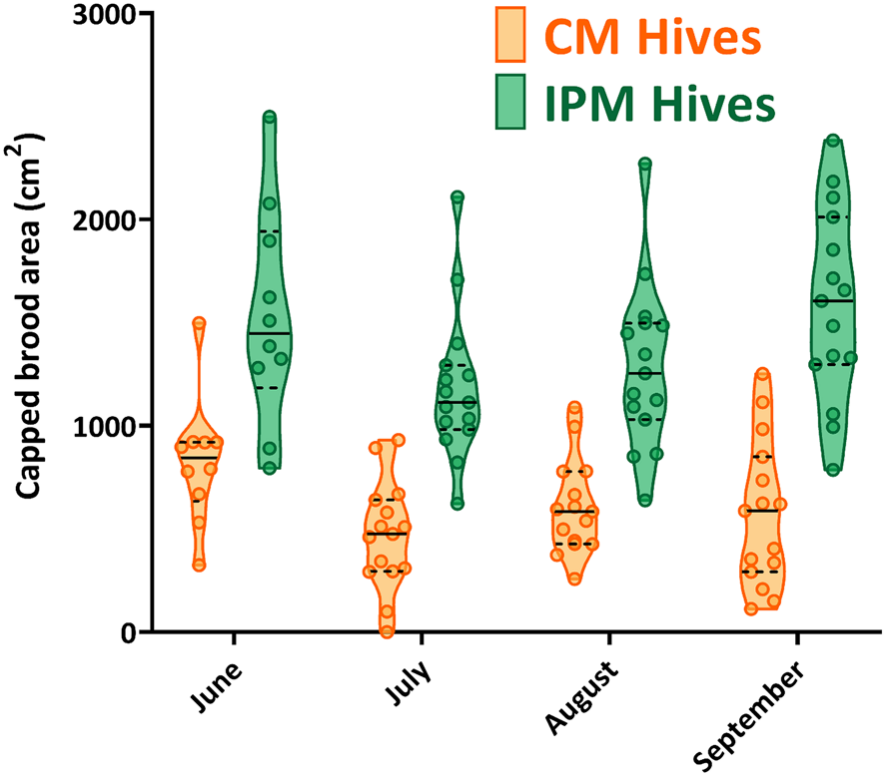
The area devoted to capped brood was higher in honey bee colonies within IPM fields. Points in the violin plots were the average area of capped brood from the front and back of 4 frames within each hive body. Each point is the mean value of total brood area from two colonies at each site (n = 5) from 2018 to 2020 from July, August, and September, and only 2019–2020 in June. Solid lines within violin plot represent 50th percentile (median), with lower and upper dashed lines indicating 25th and 75th percentiles, respectively.

Across all three experimental years, honey bee colony weight gain was 80% higher (*P* < 0.001) in IPM (30.09 ± 1.96 kg) than CM (16.70 ± 1.90 kg) colonies (Fig. 1A, Table S2A for statistical model for all honey bee metrics). Similarly, the peak measurement of growth was significantly higher (*P* < 0.001) in IPM (36.22 ± 1.97 kg) than CM (24.81 ± 1.91 kg) colonies. Immature bee populations, measured through area devoted to capped brood, was 132% higher (*P* < 0.001) in IPM colonies (1377.4 ± 61.09 cm^2^) than CM (592.84 ± 62.57 cm^2^) (Fig. 2). Repeated measures analysis showed that metrics of colony growth varied significantly over the year (F_9,252_ = 59.44, *P* < 0.001; F_3,54_ = 7.88, *P* < 0.001 for weight and capped brood, respectively). There was also an interaction between treatment and time of year for colony weight (Fig. 1; *P* = 0.013) that was driven by increasing differences over time, but no such relationship for immature bee populations (*P* = 0.493).

### Varroa mite levels were unaffected by pest management system, but IPM hives had lower mortality and greater overwintering success

**Figure 3:**
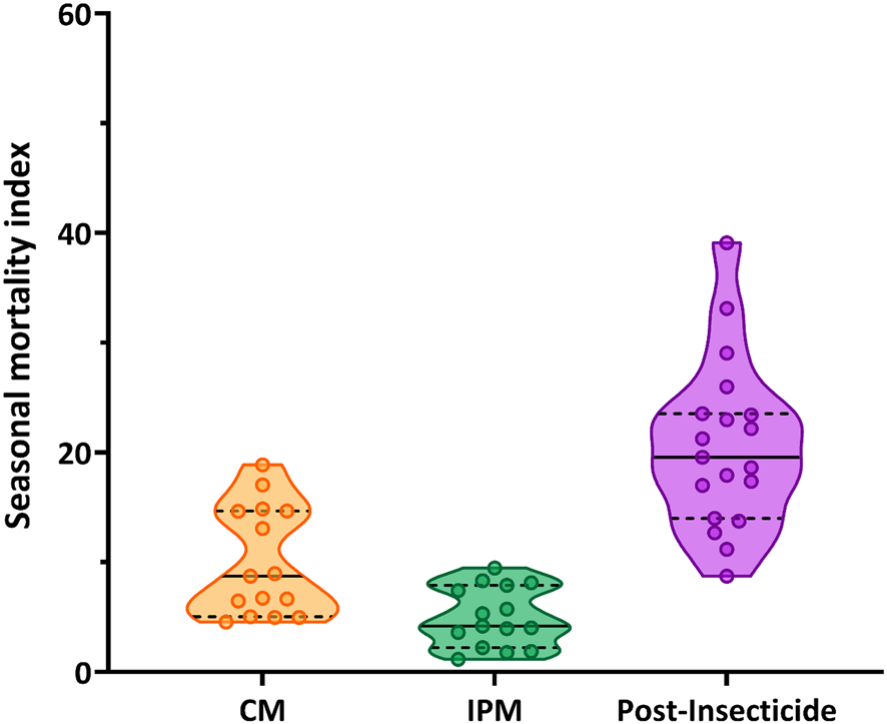
Seasonal average of at-hive mortality was highest following insecticide sprays to watermelon. Points in each violin plot were the seasonal mean of at-hive mortality measured using a collection board in front of each hive. Each point is the average from all hives (n = 2) at each of the five experimental sites from each treatment from 2018 to 2020. The purple post-insecticide treatment represents the two mortality counts that followed any pyrethroid foliar application from both the CM (n = 15) and IPM (n = 4) treatments. Solid lines within plot represent 50th percentile (median), with lower and upper dashed lines indicating 25th and 75th percentiles, respectively.

The varroa mite infestation rate throughout the season did not differ (*P* = 0.166) between CM (3.77 ± 1.02 mites per hive per season) and IPM (1.26 ± 0.34 mite per hive per season). There was an increase in mites across the season with 0.1%, 0.27%, and 0.64% infestation rates in late July, August, and September, respectively. At hive mortality was significantly different among CM, IPM, and post-insecticide treatments (*P* < 0.001). IPM (5.02 ± 2.62 bees/hive), CM (10.02 ± 4.81 bees/hive), and post-insecticide (20.6 ± 7.44 bees/hive) were all different from one another based on post-hoc comparisons (Fig. 3). Successful overwintering occurred in only 10% of colonies from CM fields compared to 57% survival from IPM hives.

### Bumble bee colonies grew larger and were more reproductively successful in IPM fields

The final weight change of *B. impatiens* was significantly higher (*P* < 0.001) in IPM (63.38 ± 6.98 g) than in CM (− 45.13 ± 7.69 g) colonies, which averaged a decline in weight over the 6 weeks in the field (Table 1, Fig. 1B; Supplementary Table S2B for statistical models for all bumble bee metrics). This weight change was reflected in worker bee count (CM: 31.00 ± 4.18, IPM: 55.93 ± 5.61) and the total worker weight (CM: 3.44 ± 0.49 g, IPM: 6.65 ± 0.89 g), which were significantly higher in IPM colonies at *P* < 0.001 and *P* = 0.001, respectively. Trends of more robust colonies in IPM fields remained consistent with higher queen weight (*P* < 0.001), live queen counts (*P* = 0.001), larval counts (*P* = 0.014), egg counts (*P* = 0.002), worker honeypot counts (*P* = 0.028), and total cell counts (*P* = 0.003) compared to CM colonies (Table 1). CM colonies also had more than twice as many dead workers (57.52 ± 7.46) than the IPM (27.35 ± 2.95) colonies.

**Table 1:** Performance variables measured from dissection of *Bombus impatiens* colonies after 6-week placement in experimental fields. Each column represents experimental years (2018–2020) and whether the colonies were placed within a conventional management (CM) or an integrated pest management (IPM) system. Each value is the average (± SEM) from four colonies placed inside each of the ten experimental fields.

*B. impatiens* hive variables	2018		2019		2020	
	CM	IPM	CM	IPM	CM	IPM
Colony weight change (g)	− 16.05 ± 3.05	35.9 ± 2.81	− 56.6 ± 8.18	69.4 ± 4.65	− 62.75 ± 6.27	84.85 ± 5.66
Queen weight (mg)	292.81 ± 36.97	687.89 ± 87.61	158.21 ± 39.34	554.76 ± 48.82	308.45 ± 65.58	522.39 ± 41.18
Worker weight (mg)	1762.99 ± 199.16	4334.01 ± 366.07	4652.15 ± 330.47	10,297.13 ± 954.38	3909.56 ± 399.19	5305.3 ± 317.3
Worker count (no. hive^−1^)	18.8 ± 1.7	51.95 ± 3.87	33.7 ± 14.1	77.3 ± 7.02	42.75 ± 4.36	48.55 ± 2.09
Total cell count (no. hive^−1^)	188.6 ± 9.68	257.6 ± 8.97	194.2 ± 9.07	296.45 ± 13.59	259.4 ± 16.51	326.35 ± 20.04
Worker honeypots (no. hive^−1^)	66.3 ± 7.13	87.6 ± 9.59	59.2 ± 5.55	133.35 ± 10.29	82.6 ± 7.29	89.3 ± 7.03
Worker larval cells (no. hive^−1^)	18.6 ± 1.87	23.7 ± 3.37	20.55 ± 2.3	30.25 ± 3.51	24.95 ± 3.13	46.25 ± 4.8
Live eggs (no. hive^−1^)	6.1 ± 2.19	23.61 ± 3.41	13.7 ± 2.51	63.05 ± 13.27	5.75 ± 1.85	31.2 ± 3.95
Dead worker count (no. hive^−1^)	58.9 ± 6.11	34.6 ± 2.22	60.75 ± 5.52	20.3 ± 2.28	52.9 ± 9.28	27.15 ± 2.99

### IPM fields contained a larger and more diverse pollinator community

**Figure 4:**
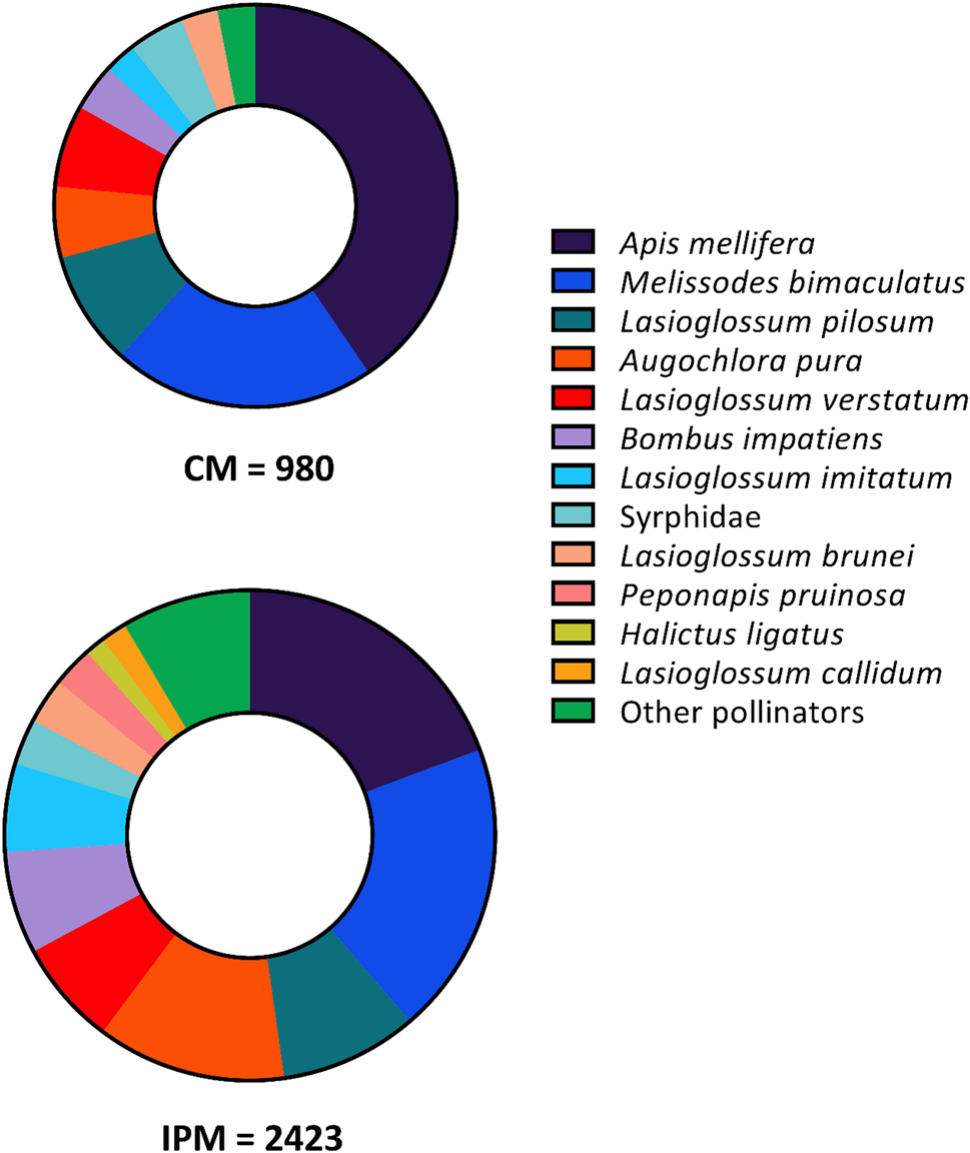
Pollinator community was less abundant and species rich in CM fields (top) compared to IPM fields (bottom). Each doughnut chart shows collected pollinators from surveys of watermelon flowers during bloom from 2018 to 2020 (1125 total sampling minutes for each graph). Graphs are scaled to the proportion of pollinators observed between the two fields and colors represent pollinators identified to the lowest taxonomic unit possible. Any species that represented ≤ 1% of either system’s community was grouped into the “other pollinators” category. See Supplementary Table S3 for raw data on counts for all species.

**Figure 5:**
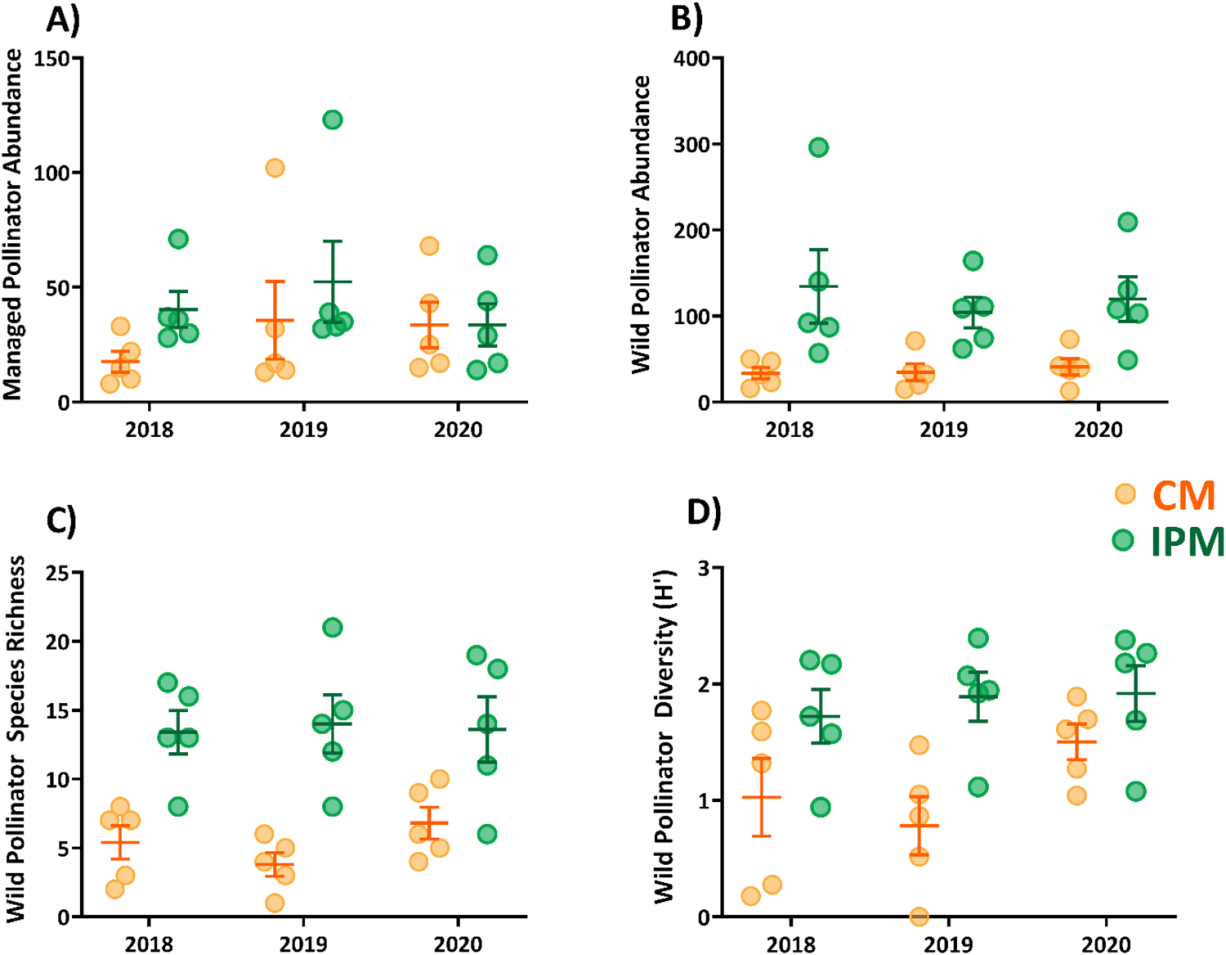
Pollinator communities with managed species separated to show abundance of managed colonies (**A**) and wild species (**B**) observed actively visiting watermelon flowers. To demonstrate the effect of managed species to community metrics managed species were removed from calculations of both species richness (**C**) and Shannon H′ diversity (**D**). Each point within a cluster (n = 5) represents 5 weekly collections during that field season (75 total minutes). Whiskers within the plot show the average ± SEM of all sites within each cluster.

There was a total of 4909 pollinators from 41 morphospecies collected from experimental fields over the three-year period (Fig. 4; see Supplementary Table S2C for statistical summary of wild pollinators and Supplementary Table S3 for raw data across all species). The most abundant species were honey bee (n = 1381), *Melissodes bimaculatus* (n = 997), *Lasioglossum pilosum* (n = 469), and *Augochlora pura* (n = 389). The pollinator community was dominated by wild species (n = 3235 specimens) compared to managed species (n = 1674 specimens). This difference was more pronounced in IPM fields; managed pollinators represented 44% and 26% of the collection from the CM and IPM fields, respectively. Despite being a relatively smaller proportion of the pollinator population, the seasonal abundance of managed pollinators was 37% higher in IPM (42.13 ± 6.71) than in CM (28.93 ± 6.36) fields (Fig. 5A). Similarly, the number of wild pollinators collected was 107% higher (*P* < 0.001) in IPM (119.4 ± 16.10) than in CM (36.4 ± 4.61) fields (Fig. 5B). Species richness was similarly 128% higher (*P* < 0.001) in IPM (15.67 ± 1.38) than in CM (6.87 ± 0.65) fields (Fig. 5C). While there was no effect of management system on species evenness (J′) (*P* = 0.958), pollinator communities were more diverse (Shannon H′) (*P* < 0.001) in IPM (2.03 ± 0.09) than in CM (1.37 ± 0.11) fields (Fig. 5D).

### Neonicotinoid residues were detected more frequently in managed colonies from CM fields

Honey bee comb from IPM fields contained at least one neonicotinoid in 63% of samples, while all CM hives contained residues of at least one neonicotinoid product at high enough levels to be quantified (Table 2A). Imidacloprid was significantly higher (*P* = 0.005) in CM (1.78 ± 0.33 ng/g) than in IPM hives (0.21 ± 0.06 ng/g) (see Supplementary Table S2D for statistical models for all residue variables). Similarly, both clothianidin (*P* < 0.05) and thiamethoxam (*P* < 0.001) were significantly higher in CM than IPM hives. Bumble bee nest material contained at least one neonicotinoid in 97% and 13% of samples from CM and IPM fields, respectively (Table 2B). There were significantly higher residues of imidacloprid (*P* < 0.001) in CM (0.46 ± 0.01 ng/g) than in IPM (0.02 ± 0.01 ng/g) hives. While the average concentration of clothianidin (*P* = 0.007) was higher in CM hives, there was no difference in thiamethoxam from IPM hives. Detection of all non-neonicotinoid pesticides was similar between treatments (Supplementary Table S4).

**Table 2:** Neonicotinoid insecticide residues in both honey bee comb (A) and bumble bee nest material (B). Any sample below the limit of detection (0.0275, 0.0235, and 0.0056 ng/g for clothianidin, imidacloprid, and thiamethoxam, respectively) was considered the minimum value.

A	Neonicotinoid residue from honey bee colonies					
	Conventional			IPM		
Year	Percent detection (10)	Median (ng/g)	Range (ng/g)	Percent detection (10)	Median (ng/g)	Range (ng/g)
*Imidacloprid*						
2018	100%	1.07	0.325–3.28	40%	< LOD	< LOD-0.64
2019	90%	1.06	< LOD-3.73	40%	< LOD	< LOD-0.44
2020	100%	2.39	0.58–9.08	50%	0.12	< LOD-1.36
*Clothianidin*						
2018	100%	1.12	0.48–2.24	20%	< LOD	< LOD-0.64
2019	100%	1.63	0.36–2.52	40%	< LOD	< LOD-1.06
2020	80%	1.41	< LOD-2.83	30%	< LOD	< LOD-0.86
*Thiamethoxam*						
2018	40%	< LOD	< LOD-3.01	0%	< LOD	< LOD
2019	20%	< LOD	< LOD-2.42	0%	< LOD	< LOD
2020	90%	0.26	< LOD-0.90	20%	< LOD	< LOD-0.23

B	Neonicotinoid residue from bumble bee colonies					
	Conventional			IPM		
Year	Percent detection (10)	Median (ng/g)	Range (ng/g)	Percent detection (10)	Median (ng/g)	Range (ng/g)
*Imidacloprid*						
2018	70%	0.21	< LOD–0.46	0%	< LOD	< LOD
2019	70%	0.25	< LOD–0.66	20%	< LOD	< LOD–0.36
2020	100%	0.89	0.32–2.16	0%	< LOD	< LOD
*Clothianidin*						
2018	40%	< LOD	< LOD–6.22	0%	< LOD	< LOD
2019	100%	1.7	0.32–4.34	30%	< LOD	< LOD–1.06
2020	40%	< LOD	< LOD–1.74	0%	< LOD	< LOD
*Thiamethoxam*						
2018	0%	< LOD	< LOD	0%	< LOD	< LOD
2019	0%	< LOD	< LOD	0%	< LOD	< LOD
2020	30%	0.26	< LOD–0.90	0%	< LOD	< LOD

## Discussion

Our data show that a conventional approach to pest management has a negative effect on the health of managed bees and reduces the abundance and diversity of wild pollinator visitation. Importantly, significant hazards to pollinator abundance and performance were observable in the first year of the experiment, i.e., the negative effects from implementation were easily seen in conventional fields when compared to the paired IPM field. This outcome stands in contrast with other pollination enhancement strategies such as wildflower strips that can take several years or longer to generate measurable effects^62^. In a pollinator-dependent crop such as watermelon the health of insects supplying pollination may be as essential to consider as the health of the plants themselves. At typical stocking rates of 1–2 honey bee hives per acre for cucurbit crops, the rental cost for a single 20 acre watermelon field ranges from $1,755 to $2,510 (average-sized field^63^, adjusted for inflation from most recent 2017 data)^64^. With such a high investment cost there is a clear benefit to maximizing honey bee colony growth and flower visits while they are in the field and maintaining positive relationships with the beekeeper supplying them. Outside of this annual cost of commercial colonies (both honey bee and bumble bees), the conservation of wild bees in these crops may be equally, or more, important for ensuring adequate pollination is reached. If IPM practices can continually be implemented, wild bee populations could reduce the number of required managed pollinator colonies and potentially provide additional savings to the farmer.

In all experimental years, honey bee colonies in the IPM fields were more productive at accumulating food resources and producing new brood than CM fields. End of the season weight gain was nearly twice as high in IPM fields, indicating that IPM colonies collected pollen and nectar at a much higher rate. Growth patterns of IPM colonies were similar to previous examples in agricultural landscapes (Iowa soybean fields^9^), i.e., high growth during crop bloom and a leveling off or decline in weight at the end of summer. The overall colony growth was likely facilitated by the larger areas of capped brood in IPM hives. Although the images analyzed are only a fraction (~ 25%) of the hives total available space for egg laying/larval development, this snapshot of reproductive growth over time demonstrates a significantly higher amount of capped brood after as little as two months in the different environments. IPM hives with higher weight, worker numbers, and increased resources represent a colony that is more likely to successfully overwinter or provide a beekeeper with a usable hive for the following season^65^.

The lack of a fully factorial design in this experiment, to replicate grower practices in both crops, did not allow us to differentiate the relative effects of the neonicotinoid insecticides used in either corn or watermelon or the foliar pyrethroid applications to the watermelon crop. The quantification of neonicotinoids provides some idea of which products are accumulating in the colonies, but we cannot be certain what portion of this exposure came from outside of the experimental arena but still within the range of foraging. The at-hive mortality index measurement demonstrates some of the effect the pyrethroid sprays have on honey bee colonies. The counts of dead bees following an insecticide spray were 99% and 310% higher than CM and IPM counts, respectively. This increase in mortality after an insecticide spray, even in CM fields with higher baseline mortality counts, reveal that in-season foliar insecticide applications directly impact pollinator health, even when applied in late afternoon/evening hours to minimize the likelihood of honey bee exposure. A previous experiment^66^ similarly found that applications of the pyrethroid insecticide lambda cyhalothrin in combination with exposure to neonicotinoids increased honey bee worker mortality. Synergism between pesticides has been reported at colony and landscape scales^44,67,68^, and could contribute to managed bee colonies in the CM fields experiencing significant reductions to growth and health. Instead of looking at the effect of the removal of a single product or active ingredient, the IPM system we used here represents the simultaneous removal or reduction of several products across both the corn and watermelon crop. New honey bee colonies were used each year and the lack of initial food resources compounded with the negative impacts of foraging on cucurbits^69^ may be additional stressors to colonies that lead to the strong negative effects in CM colonies. Conversely, the use of new colonies and frames each experimental year ensured a more consistent starting point across all colonies and minimized previously acquired pesticide residues. While there are examples of honey bee colonies unaffected by field-relevant neonicotinoids^36,70^, pesticide synergy may have led to pronounced negative effects in CM colonies.

Bumble bees experienced similar negative effects to colony growth when placed in a CM compared to an IPM system. Popularity of bumble bees has increased with colonies sourced from commercial insectaries placed in the field to augment pollination during crop bloom^71^. *B. impatiens* is native to the eastern U.S. and, coupled with their growing popularity as a commercial pollinator, makes it useful as an additional social pollinator species to monitor. To deploy *B impatiens* colonies as quickly as possible there was no formal assessment on the initial heterogeneity of bumble bee nests (outside of ensuring all colonies were active), however initial weight assessments (Fig. 1B) showed that the total colony weights at deployment in the field were not different from one another. The decreased bumble bee in-field abundance and colony fitness in the CM fields is a clear indication of the hazards of insecticide use to bumble bees. The negative effects of neonicotinoid exposure are well documented in bumble bees^45,72,73^, but these studies are often conducted in a controlled laboratory or enclosed greenhouse environment that eliminate or restrict the ability to forage. A field study found that a related bumble bee, *B. terrestris*, in crops treated with neonicotinoid and pyrethroid seed treatments experienced reduced growth compared to colonies in untreated fields^36^. Colony dissection allows for an examination of the effect of insecticide exposure on resource gathering and reproductive development^73,74^. A lower number of eggs, larvae, workers, and queens in CM compared to IPM colonies demonstrates that higher frequency insecticide use created an environment hazardous enough to lead to deleterious effects to colony growth.

Despite our observation of fewer honey bee and bumble bee foragers in CM fields, samples from both treatments consistently contained neonicotinoid residues from both crops. Neonicotinoid residues have been found in the soil and pollen in row crop^32,75^ and cucurbit^76–78^ fields. These residues accumulating in managed colonies has been previously documented^26^, but this study identifies that IPM adoption was sufficient in reducing the concentration and detection frequency of insecticide residues within the colony of both pollinators. In many cases the range of values in CM colonies were well above documented oral LD_50_ values for pollinators^79,80^. An important context for these values is that it requires direct feeding by bees within the colony. Beebread from honey bee colonies is a source of food for larvae and adults, but enough would have to be eaten to result in lethal or sublethal effects^38,81^. A previous survey of honey bee wax detected several fungicides and pesticides at high levels (~ 1 µg/g); these detections are far higher than any in this experiment, and likely represent an accumulation of residues over multiple years of exposure.

The difference in managed/wild pollinator communities between treatments demonstrate that a conventional approach to pest management more strongly affects wild pollinators and increases the reliance on managed bees to provide pollination. The average species richness in IPM sites was 128% higher than CM sites, demonstrating a favorable environment for wild pollinators. Wild species are more effective pollinators than honey bees; in an experiment using watermelons in Florida, *Melissodes, Bombus,* and *Agapostemon,* all deposited similar or greater amounts of pollen to watermelon flowers compared to honey bees^41^. These three genera were among the most abundant, and all were more common in IPM than CM fields. In commercial pumpkins (a related cucurbit crop) in Pennsylvania there was a similarly diverse community of 37 species foraging on flowers with most visits (> 78%) coming from honey bees, *B. impatiens,* and the cucurbit specialist squash bee *Peponapis pruinosa*^82^. In organic cucumber fields in Indiana, 28 bee species were reported, but honey bees were the most abundant species with 66% of visits^83^. A comparison of pollinator communities in Midwest pumpkin, cucumber, and watermelon fields similarly found that the watermelon pollinator community was more diverse and less reliant on managed species^44^. In this comparative study on commercial farms, cucumbers were nearly entirely visited by honey bees (98%) with pumpkins and watermelon at 41% and 42%, respectively. It is impossible to determine whether collected *B. impatiens* were from a wild or commercial colony, therefore collected specimens were considered “managed”, meaning the proportion of wild species in the watermelon pollinator community is likely even larger. The wild bee community found in this experiment was primarily Halictidae spp. and *Melissodes bimaculatus*. A consistent trend across these experiments was the strong negative effect of CM vs IPM treatment on wild bee abundance and diversity in agricultural fields.

While the wild pollinator communities were different between treatments in the first year of collection during watermelon bloom, it is important to consider that the previous field season the planting of corn (as well as the concurrent surrounding corn crop) was a potential exposure for the CM treatment before any pollinator data were collected. This additional season of possible exposure to contaminate non-target flowers or soil could have caused a lower existing community before watermelon crop blooming began. The accumulation of neonicotinoids, as systemic insecticides, over multiple seasons at the CM field locations could lead to strong effects that have been observed in other studies^31,36,84^. Pyrethroid applications are known to have a repellent effect^85^ on insects, therefore it is possible that wild pollinators chose to avoid contaminated fields when not directly exposed at lethal levels. Additionally, it is important to acknowledge the likely impact that the surrounding landscape had on the pollinators observed in this study. A limitation of our data is its focus on the local effects of crop management and not taking a landscape perspective. While watermelon flowers served as the focal resource to both managed and wild pollinators, they undoubtedly visited additional floral resources during and outside of watermelon bloom. The foraging range of pollinators extends far beyond the spatial domain of the experimental sites and small variations to that surrounding landscape likely further influence pollinator health^49–51^. Non-crop flowers, such as agricultural weeds, are a significant resource to pollinators and in agriculturally-dominated landscapes they serve as an additional source of insecticide exposure, even to pollinators in the IPM fields that were free of such products^28^. It is difficult to isolate which possible route of insecticide exposure (corn NST, watermelon soil drench, pyrethroid sprays, or potential exposure from outside the study area) is responsible for proportion of insecticide hazard, but the purpose of this study was to replicate the settings of commercial agriculture and mimic the complete suite of insecticide exposures that pollinating insects, both managed and wild, would experience.

## Conclusion

Indiana is routinely among the top five watermelon producing states, with neighboring states similarly leading the nation in other cucurbit crops^86^. These systems are often rotated with and surrounded by row crops that nearly always use NSTs regardless of regional pest pressure^87^. The use of NSTs combined with the foliar insecticides in cucurbits^44^ leads to high environmental stress on pollinators within these agroecosystems. Frequent within- and extra-field insecticides in a crop reliant on pollinators can threaten pollinator health, in turn compromising yield. This experiment demonstrates how simple changes to insect pest management—namely, the adoption of a previously developed, scouting-based IPM program—can improve the health of managed colonies and the entire pollinator community. These changes to pest management are well-established as proven practices for decades. Some of the most influential factors in grower decision making, cost and ease of implementation^88^, can be addressed through this work and provide growers a compelling piece of evidence that IPM can be successfully put into practice.

Wild pollinators are more sensitive to environments with high insecticide use^89^ and their increased abundance, richness, and diversity in IPM compared to CM fields in this system support earlier observations. The recruitment and retention of wild pollinators represents a potential increase in pollination services that are “free” to growers, and lead to increased fruit quality and weight. Removing prophylactic insecticides such as seed treatments that provide negligible yield improvements^53,54,90^, or unnecessary calendar-based foliar sprays can be an important step in improving conditions for pollinators and maximizing the opportunity to realize the yield benefits they provide.

Collectively, the findings from this experiment provide evidence that commitment to IPM can provide an environment more suitable for pollinators. Maintaining food security and minimizing environmental degradation are often overlapping concerns and sometimes conflict with one another. However, these experiments demonstrate that those goals are not mutually exclusive, and less intensive insect control via IPM provides an opportunity to conserve essential pollinators while still providing growers with the tools to manage key insect pests.

## Materials and methods

### Experimental design

This four-year experiment took place from 2017 to 2020 on five of the Purdue Agricultural Center (PAC) research farms across Indiana, USA. At each site, two fields (separated by 4.63–6.63 km; average 5.6 km) were randomly assigned to either a conventional management (CM) or integrated pest management (IPM) program. Regardless of treatment, all fields had the same crop arrangement: the entire area (4.8–7.7 ha) was planted with corn, except for 0.2 ha of watermelons embedded within the corn matrix, surrounded on all four sides. The two treatments differed only in insecticide inputs; all other management practices (e.g., tillage, fertilizer, herbicides/fungicides) were standardized for each pair of sites. For additional detail on site history, land use, and crop management, previous work can be referenced^78^. Corn was planted in all 4 years of the study (beginning in 2017), whereas watermelon started 1 year later (2018). The purpose of this staggered start date was to allow the first year for corn to impose initial treatment differences in insecticide use that carryover to subsequent years. Thus, during the initial year of watermelon-pollinator surveys, ground-nesting bees were potentially exposed to soil residues from the prior year’s corn crop.

CM fields mimicked the insecticide inputs typical of Indiana row crop and vegetable production. Corn seed (var. Spectrum 6334) was coated with thiamethoxam (Cruiser 5FS @ 1.25 mg a.i. per seed), one of the most widely used neonicotinoid products by US farmers. Transplanted watermelons (var. ‘Fascination’) received imidacloprid (Wrangler^®^ @ 814.09 ml/ha) as a soil drench. While not as ubiquitous as NST in corn, neonicotinoids applications as a tray drench as seedlings or soil drench at transplanting are common practices^63^. Additionally, CM watermelons were sprayed with the insecticide lambda-cyhalothrin (Warrior II^®^ pyrethroid @ 140.3 ml/ha) via tractor-drawn air blaster or boom sprayer at 4, 6, 8, and 10 weeks post-transplant, resulting in four foliar applications each season. These sprays were made as late in the day as possible to avoid peak bee foraging times, with a majority of applications taking place later than 17:00. The CM insecticide program is based on prior on-farm surveys of Indiana watermelon growers and thus replicates a typical spray regime, consisting of *ca.* five applications per season with neonicotinoids and/or pyrethroids^63^.

In the IPM system, corn seed was left untreated, except for fungicides, which were coated on seeds in both treatments (Maxim Quattro: Azoxystrobin 2.5 µg; Fludioxonil 6.5 µg; Mefenoxam 5 µg; Thiabendazole 50 µg of a.i. per seed). Similarly, IPM watermelons received no insecticides, except if the primary pest—striped cucumber beetle (*Acalymma vittatum*)—exceeded its economic threshold of 5 beetles per plant during weekly scouting^91^. When pests crossed their threshold, we applied a foliar spray of lambda-cyhalothrin, as described above. However, this only occurred four-times across the 15 site-years; once each in 2018 and 2019 and twice in 2020. None of the IPM fields were treated more than once in a growing season.

### Honey bee colony establishment

Colonies of honey bees (*A. mellifera*) were regionally sourced from Bastin Honey Bee Farm LLC (Knightstown, IN, USA). In 2018 and 2019, 2.7 kg packages with mated queens were used, while poor weather conditions in 2020 forced the use of nucleus (nuc) hives that were modified to have reduced food stores/capped brood and an increased number of bees to mimic the packages used in earlier years. Bees were housed in pre-weighed 8-frame Langstroth hives with plastic foundation frames (#HK-560 Hackensack, MN, USA). Hives were only used once per year of the experiment and later replaced, i.e., we did not place the same hive out in multiple years so that each unique year-site was not confounded by conditions experienced in prior years of the study.

Each field received two hives, placed at opposite corners in the space at the transition between the watermelon and corn crops, in an arrangement to avoid farm management (e.g., driving lanes, irrigation). This design also prevented hives from being directly sprayed with insecticide and the stocking rate was within the recommended range of 1–5 colonies per acre^92^. Once purchased, all colonies were installed and placed within a three-day period: 9–11 May 2018, 2–4 May 2019, and 19–20 May 2020. In 2018 and 2020, all corn was planted prior to hive placement; however, in 2019 weather conditions delayed corn planting and thus all colonies were already established, and the hive entrances were blocked during the field planting (Supplementary Table S1). Establishment was confirmed by observing new eggs or larvae in frames. Only one package did not have a viable queen (2019) and after replacement 2 days later, successful eggs were observed. Colonies remained in the field until late-September or early-October (spanning the full management periods for both crops, except for corn harvest), after which they were overwintered in an apiary yard within Martell Forest located outside of West Lafayette, IN. In the apiary, all colonies were provided with supplemental sugar solution (1:1 ratio sucrose: water) prior to temperatures dropping consistently below 0 °C. In the following spring, hives were checked for successful overwintering and recorded as either alive or dead. Surviving colonies were removed from the hive boxes and all frames were replaced prior to the next field season when a new set of colonies were used.

### Honey bee colony growth

Colony size is one of the strongest predictors of overwintering success^65^, brood production^93^, and weight of accumulated foraging resources^65^. After placement into each pre-weighed hive box, colonies were weighed (Doran 7400, Doran Scales Inc., St. Charles, IL, USA) to calculate initial weight and then reweighed approx. every two weeks until hive removal from the field (10–11 measurements per hive per year). When colony inspections showed brood production in more than half of upper box frames (typically late June), two honey supers were pre-weighed and added to all colonies to allow additional resource storage throughout the season. The pre-weight measurements of hive boxes and supers were subtracted from colony weight measurements to accurately quantify colony population and resource gathering.

Successful development of new brood represents a greater number of bees within the hive to transition to a foraging role for pollen/nectar gathering and capped brood (pupation) allows for a standardized timepoint to measure the production of new brood in a colony^9,30^. As an additional measure of colony growth, photographs of frames were taken to quantify capped brood^94^. Photographs were taken from a subset of frames from every hive monthly from July–September in 2018 and June–September in 2019 and 2020. Frames inside the second hive body were organized by labeling all frames 1–8 with the first being the northmost frame. During picture sessions, each side of frames 2, 4, 6, and 8 were brushed free of bees, photographed, and returned, maintaining the same frame order and orientation within the hive body. All hives had pictures taken within one week of one another for each month’s sample. Individual images (n = 1600) were opened in Microsoft Paint (Microsoft Corporation, Redmond, WA) and all cells in the frame dedicated to capped brood were filled in with the same color. Colorized frame photos were analyzed using ImageJ (US NIH, USA) to quantify the area of each frame dedicated to capped brood within each colony.

### Honey bee mortality

While most of the honey bee mortality (80–98%) occurs away from the hive (Johansen and Mayer 1990, Porrini et al. 2002), the cleanliness behavior within the hive allows for at-hive mortality to serve as a proxy or “mortality index” for comparisons across hives^30,34^. Plywood boards (1 m × 1 m with 5 cm raised edges on all sides) were treated with a white paint/stain and placed directly in front of each colony to collect dead bees from within the hive that were removed by other members of the colony. The number of dead or dying (categorized by spasms or twitching behavior when prodded) individuals on the board was measured each Monday, Wednesday and Friday and summed for a weekly total mortality. Multiple within-week measurements were used to reduce the number of dead bees lost due to scavenging from small mammals or birds. For each count, the board was removed from in front of the hive to a safe distance to count all bees. Dead individuals were removed along with any detritus and the board was replaced in front of the hive. Mortality was measured for the duration of colony placement in the field, ending the week prior to overwintering.

### Varroa mite counts

Although each colony was newly established, we counted varroa mites to observe any first-year accumulations. Counts were conducted three times each year, mid-July, early August, and mid-September, using a Varroa Easy Check (Véto-pharma, Palaiseau, France) container to evaluate colony mite levels. The bottom collection portion of the container was filled with ethanol to the point where it was nearly touching the inner collection cup. Then, using a bee brush (#M00751, Dadant, Hamilton, IL, USA) approx. 300 bees (1/2 cup) from a frame containing brood were placed into the inner collection cup and the lid was secured immediately to prevent escaping bees. The container was shaken for 60 s to kill all bees and any mites on them, which fall through holes in the inner collection vessel. The transparent outer bowl allowed for mite counting, and then the entire container was emptied and washed once with water to remove all remaining bees or mites prior to the next mite count. The number of counted mites was divided by three to calculate the percent infestation of the hive.

### Bumble bee colonies

Colonies of bumble bees are becoming a popular alternative to honey bees as an option to provide managed pollination services in watermelon due to their success in enclosed environments and efficiency as pollinators in diverse crops. The common eastern bumble bee (*Bombus impatiens* Cresson) is a native species within the study region and the most common managed bumble bee species available for the eastern United States. A Quad colony (Koppert Biological Systems, Howell, MI, USA) containing 4 separate *B. impatiens* colonies was placed in each field 4–5 weeks following watermelon transplant to synchronize colony growth with the crop bloom period. At placement, each individual colony was labeled and weighed (Tayler Precision Products TE22FT, Capacity 10 kg × 1 g) and left in the space between corn and watermelon crops under a tarp for protection from rain and sun. Each week, the entrance to the colonies was temporarily altered such that foraging bees could return but new foragers could not leave. After *ca.* 1 h in this condition, colony weight was measured and the entrance was reopened. This process was repeated for 6 weeks, after which colonies were placed in a − 20 °C freezer to kill all remaining bees and preserve the colony for later inspection.

After at least 5 days in the freezer, colonies were dissected in the lab. We recorded the number and weight of all workers, males, and queens that were still alive at the time of freezing, along with counts of already dead bees (i.e., died prior to freezing). The two groups were distinguishable based on appearance and location—dead bees showed clear signs of decay and were often found on the edges of the colony away from the nest material. Additionally, we recorded the number of constructed cells with nectar resources, eggs, larvae, and pupae of both worker and queen types (distinguished by size). These caste performance metrics, coupled with colony weight change, informed the condition and health of the colony in either treatment group.

### Wild pollinator communities

Weekly collections of insect visitors to watermelon flowers were conducted at all sites to measure the community of wild bees and other taxa contributing to crop pollination. Collections began around 6 weeks post-transplant of watermelon seedlings and ended after completion of 5 consecutive weekly surveys; this was typically from late June to early August and took place to coincide with watermelon bloom based on transplanting date. Collections took place on the same date at each pair of fields per site to account for daily or weekly differences in weather conditions affecting pollinator foraging activity. Sampling occurred between 9:00 and 13:00 with low cloud cover, wind speeds < 16 km/h, and temperatures between 15 and 32 °C. This weekly collection extended through the peak blooming period of watermelon when most pollination occurs.

Pollinators were collected with a hand-held insect vacuum (Bioquip #2820GA) with a collection chamber to capture insects visiting watermelon flowers. Sampling occurred along a transect extending from the field edge and walking between plant rows for a 15 min period, collecting all insects actively visiting watermelon flowers. This sampling time typically allowed for the entire field to be surveyed. The process resulted in 75 min total collection time per field per year (= 15 min weekly transect × 5-week duration). Upon completion, the collection chamber was removed and placed in a cooler until returning to lab where it was stored at − 20 °C until later identification. All pollinators were pinned and identified to the lowest taxonomic level. Most specimens were identified to species, except for hoverflies (Syrphidae) and several *Lasioglossum* sp. (Halictidae) that were identified to morphospecies. Bee specimens were identified using the taxonomic keys^95–97^.

### Pesticide residues

Samples from within both the honey bee and bumble bee colonies were taken in each experimental year. Wax from each honey bee colony was collected during the first watermelon harvest, at which point all insecticides in the crop had been applied and hives are often removed in commercial operations. A sample of approximately 10 g of wax was taken from the 5th frame of the top hive body from a section of the frame free of observable eggs or brood. Samples were immediate placed in a cooler and stored in a − 20 °C freezer until processing. During the bumble bee colony dissections, approximately 5 g of nest material (open and nectar-containing cells) was collected into small freezer bags and placed in a − 20 °C freezer. When processing began, each wax or nest material sample was finely ground in liquid nitrogen using a mortar and pestle until the sample was reduced to a powder and a 0.5 g aliquot was used for the residue analysis. All materials were sanitized with ethanol between each sample to avoid contamination.

Processing methodology for samples followed a modified QuEChERs protocol for residue quantification optimized for the high-lipid matrix of the wax/nest material^26,73^. For bee bread/nest material, 0.5 g of sample was mixed with extraction solution (15 ml dH_2_O + 15 ml acetonitrile) and 10 µl of internal standard solution (clothianidin-d3, imidacloprid-d4, thiamethoxam-d3, and acetamiprid-d3 at a 10 ng/µl concentration) simultaneously and vortexed. Samples were combined with 6 g of magnesium sulfate and 1.5 g of sodium acetate, inverted, vortexed, and centrifuged at 2500 r.p.m. for 10 min, after which 10 ml of the top layer of supernatant was transferred to a QuEChERS Dispersive Kit (Agilent Technologies, Santa Clara, CA, #5982-5456) and again inverted, vortexed, and centrifuged at 4000 r.p.m. for 5 min. Supernatant (6 ml) was transferred to a clean 15 ml tube and dried completely in a speed vacuum (Savant SC250EXP, Thermo Scientific, Waltham, MA). All samples were resuspended in 200 µl acetonitrile, vortexed, centrifuged, and the supernatant was transferred to 96-well plates. Immediately prior to instrument analysis, samples were re-suspended with 200 µl 50% acetonitrile dH_2_O solution.

We screened samples for the active ingredients of all fungicides and insecticides used during the experiment. Samples were analyzed via liquid chromatography and tandem mass spectrometry at the Bindley BioScience Center at Purdue University, West Lafayette, IN. An Agilent Zorbax SB-Phenyl 2.1 × 100, 3.5 µm column was used for LC separation and an Agilent 1200 Rapid Resolution LC system coupled to an Agilent 6460 series triple quadrupole mass spectrometer was used to identify pesticide residues based on retention time and co-chromatography with analytical standards of all pesticide targets. Deuterated neonicotinoids were used to quantify the concentration of neonicotinoids in samples based on the relative response value. A mix of analytical standards from all other pesticides used in the experiment were subjected to a serial dilution and analyzed on the instrument to create standard curves to quantify their concentration in each sample. This protocol prioritized the detection and quantification of neonicotinoids, which made quantification of some of the other pesticides impossible. This optimized protocol limited the detection clarity of the non-neonicotinoid pesticides, therefore non-neonicotinoid products applied to the watermelon fields were not quantified and instead reported only as a presence/absence for each sample (Supplementary Table S4).

### Statistical analysis

Statistical analyses were performed using SYSTAT 13 (SYSTAT Software, Inc; Point Richmond, CA) by creating a series of linear models for abundance of both managed and wild pollinators and performance response variables. Pseudoreplication was avoided by averaging colony parameters for multiple honey bee and bumble bee hives at each field to use field as the replicate for each treatment/year^98^. Similarly, surveys of the wild pollinator community were summed across collection dates for a single community total for each site/treatment/year. This approach resulted in 30 data points for each response variable with treatment (n = 2), year (n = 3), and site (n = 5) treated as fixed effects in the model, as well as the two-way interactions between treatment and year or site. Honey bee hive mortality model included an additional treatment “post-insecticide” that included all CM and IPM hive mortality counts during the two observation periods immediately following a pyrethroid spray to that field. For this “post-insecticide” treatment the data points were taken from hives from both CM (n = 15) and IPM (n = 4) treatments to explore the acute effect of pyrethroid sprays. Any mortality counts for a honey bee colony that were in the “post-insecticide” timing were not included in the seasonal mean for that colony that was used in the model. This resulted in each replicate representing a range of mortality counts to form the seasonal mean (ranged from 8 to 37 counts per colony, average 26.6). Post-hoc pairwise comparisons (Fisher’s LSD) were used to differentiate any factors (or interactions) that were significant (p = 0.05). Additional repeated-measures analyses were conducted with linear models on seasonal changes to honey bee colony growth (collected 10 times per hive per year), capped brood area (4 times per hive per year), varroa mite load (3 times per hive per year), and bumble bee colony growth (6 times per colony per year) across each season with treatment as a fixed effect. Data were transformed (square-root or log) as necessary to meet assumptions of normality (summarized for each response variable in Supplementary Table S2). Because insecticide data generally contain many zeroes and thus cannot be transformed to achieve a normal distribution, we analyzed pesticides using a general linear model with a binomial distribution. To do so, we converted concentration data to presence/absence based on whether any sample contained quantifiable residues over the limit of detection.

## Supplementary Information

Supplementary Information 1.

Supplementary Information 2.

## Data Availability

All data generated or analyzed during this study are included in this published article [and its supplementary information files].
