# Supplementary material for: Implementing IPM in crop management simultaneously improves the health of managed bees and enhances the diversity of wild pollinator communities
Source: Sci Rep. 2023 Jul 7;13:11033. doi: 10.1038/s41598-023-38053-5 (PMC10328965; doi:10.1038/s41598-023-38053-5)

Table S1: Information on the location and timing of honey bee colony placement and removal for all experimental years and sites. All hives within a year were placed at all sites within a 48-hour period after purchase from a local supplier.

Year	Location	Treatment	Colony ID	Field placement	Field removal
2018	TPAC Lafayette, IN	IPM	1-2	9 May	30 Sept
		CM	3-4		
	SEPAC Butlerville, IN	IPM	5-6	9 May	2 Oct
		CM	7-8		
	PPAC Wanatah, IN	IPM	9-10	10 May	1 Oct
		CM	11-12		
	NEPAC Columbia City, IN	IPM	13-14	10 May	1 Oct
		CM	15-16		
	SWPAC Vincennes, IN	IPM	17-18	11 May	3 Oct
		CM	19-20		
2019	TPAC Lafayette, IN	IPM	21-22	3 May	2 Oct
		CM	23-24		
	SEPAC Butlerville, IN	IPM	25-26	2 May	1 Oct
		CM	27-28		
	PPAC Wanatah, IN	IPM	29-30	3 May	29 Sept
		CM	31-32		
	NEPAC Columbia City, IN	IPM	33-34	3 May	29 Sept
		CM	35-36		
	SWPAC Vincennes, IN	IPM	37-38	4 May	30 Sept
		CM	39-40		
2020	TPAC Lafayette, IN	IPM	41-42	20 May	7 Oct
		CM	43-44		
	SEPAC Butlerville, IN	IPM	45-46	19 May	7 Oct
		CM	47-48		
	PPAC Wanatah, IN	IPM	49-50	20 May	6 Oct
		CM	51-52		
	NEPAC Columbia City, IN	IPM	53-54	20 May	6 Oct
		CM	55-56		
	SWPAC Vincennes, IN	IPM	57-58	19 May	5 Oct
		CM	59-60		

Table S2: General linear model output for all response variables. Significant differences are designated by bold text based on a level of $P < 0.05$ for all honey bee colonies (A), bumble bee colonies (B), wild pollinator surveys (C), and neonicotinoid residues (D). Any transformations to normalize data or separate repeated measures analyses are stated underneath response variables.

A. Honey bee colony parameters

Response Variable	Explanatory Variable(s)	df	F	*P*
**Final Weight Change**	Treatment	1,16	66.55	**< 0.001**
	Year	2,16	18.21	**< 0.001**
	Site	4,16	4.34	**0.015**
	Treatment*Year	2,16	0.47	0.633
	Treatment*Site	4,16	1.80	0.179
**Average Brood Area**	Treatment	1,16	123.11	**< 0.001**
	Year	2,16	3.99	**0.039**
	Site	4,16	0.66	0.632
	Treatment*Year	2,16	0.01	0.987
	Treatment*Site	4,16	0.23	0.917
**Varroa Seasonal Average**	Treatment	1,16	2.11	0.166
Log(x+1) transformed	Year	2,16	0.31	0.739
	Site	4,16	2.97	0.052
	Treatment*Year	2,16	0.31	0.736
	Treatment*Site	4,16	0.84	0.517
**At-Hive Mortality**	Treatment	2,28	51.12	**< 0.001**
	Year	2,28	11.79	**< 0.001**
	Site	4,28	1.43	0.25
	Treatment*Year	4,28	0.60	0.669
	Treatment*Site	8,28	0.58	0.788
**Seasonal Brood Area**	Time	3,16	9.05	**0.001**
Repeated measures	Time*Treatment	3,16	0.84	0.493
**Varroa Mite Counts**	Time	2,27	9.05	**0.001**
Repeated measures	Time*Treatment	2,27	0.84	0.493

B. Bumble bee colony parameters

Response Variable	Explanatory Variable(s)	df	F	*P*
**Colony Weight Change**	Treatment	1,16	193.22	**< 0.001**
	Year	2,16	0.13	0.880
	Site	4,16	1.58	0.228
	Treatment*Year	2,16	13.76	**< 0.001**
	Treatment*Site	4,16	0.57	0.688
**Worker Weight**	Treatment	1,16	25.98	**< 0.001**
Square root transformed	Year	2,16	15.44	**< 0.001**
	Site	4,16	2.77	0.063
	Treatment*Year	2,16	1.65	0.224
	Treatment*Site	4,16	1.09	0.393
**Queen Weight**	Treatment	1,16	72.33	**< 0.001**
	Year	2,16	1.04	0.377
	Site	4,16	1.48	0.254
	Treatment*Year	2,16	0.64	0.542
	Treatment*Site	4,16	0.81	0.535
**Queen Count**	Treatment	1,16	15.57	**0.001**
	Year	2,16	2.72	0.096
	Site	4,16	1.27	0.323
	Treatment*Year	2,16	1.13	0.348
	Treatment*Site	4,16	0.62	0.654

**Live Worker Count**	Treatment	1,16	18.25	**0.001**
Square root transformed	Year	2,16	3.94	**0.041**
	Site	4,16	0.69	0.608
	Treatment*Year	2,16	3.14	0.071
	Treatment*Site	4,16	1.30	0.313
**Dead Worker Count**	Treatment	1,16	10.64	**0.005**
Square root transformed	Year	2,16	0.63	0.548
	Site	4,16	0.40	0.805
	Treatment*Year	2,16	0.51	0.614
	Treatment*Site	4,16	0.32	0.863
**Worker Larvae Count**	Treatment	1,16	7.56	**0.014**
Square root transformed	Year	2,16	3.84	**0.044**
	Site	4,16	1.43	0.269
	Treatment*Year	2,16	1.21	0.323
	Treatment*Site	4,16	0.93	0.473
**Egg Count**	Treatment	1,16	13.34	**0.002**
Log(x+1) transformed	Year	2,16	0.79	0.473
	Site	4,16	2.38	0.095
	Treatment*Year	2,16	0.63	0.543
	Treatment*Site	4,16	0.85	0.513
**Worker Honeypots**	Treatment	1,16	5.83	**0.028**
	Year	2,16	0.63	0.547
	Site	4,16	0.52	0.725
	Treatment*Year	2,16	2.11	0.154
	Treatment*Site	4,16	0.78	0.556
**Total Cells**	Treatment	1,16	11.95	**0.003**
	Year	2,16	3.21	0.067
	Site	4,16	0.67	0.624
	Treatment*Year	2,16	0.25	0.783
	Treatment*Site	4,16	0.70	0.602

C. Pollinator Survey

Response Variable	Explanatory Variable(s)	df	F	*P*
**Pollinator Abundance**	Treatment	1,16	45.48	< **0.001**
Square root transformed	Year	2,16	0.15	0.861
	Site	4,16	8.40	**0.001**
	Treatment*Year	2,16	1.25	0.313
	Treatment*Site	4,16	0.50	0.702
**Species Richness**	Treatment	1,16	61.73	< **0.001**
	Year	2,16	0.47	0.633
	Site	4,16	4.22	**0.016**
	Treatment*Year	2,16	0.78	0.477
	Treatment*Site	4,16	0.27	0.892
**Shannon (H') Diversity**	Treatment	1,16	41.47	< **0.001**
	Year	2,16	1.58	0.236
	Site	4,16	6.43	**0.003**
	Treatment*Year	2,16	1.28	0.306
	Treatment*Site	4,16	0.26	0.899
**J' Evenness**	Treatment	1,16	0.01	0.958
	Year	2,16	0.91	0.425
	Site	4,16	2.66	0.071
	Treatment*Year	2,16	0.42	0.662
	Treatment*Site	4,16	1.21	0.343

D. Neonicotinoid residues

Response Variable	Explanatory Variable(s)	df	F	*P*
**Honey bee: Imidacloprid**	Treatment	1,16	10.89	**0.005**
Binomial distribution	Year	2,16	1.56	0.241
	Site	4,16	0.33	0.851
	Treatment*Year	2,16	2.12	0.169
	Treatment*Site	4,16	0.88	0.452
**Honey bee: Clothianidin**	Treatment	1,16	8.17	**0.011**
Binomial distribution	Year	2,16	0.17	0.848
	Site	4,16	0.25	0.905
	Treatment*Year	2,16	0.49	0.621
	Treatment*Site	4,16	1.06	0.118
**Honey bee: Thiamethoxam**	Treatment	1,16	33.34	< **0.001**
Binomial distribution	Year	2,16	6.63	**0.009**
	Site	4,16	1.17	0.362
	Treatment*Year	2,16	2.12	0.129
	Treatment*Site	4,16	0.83	0.524
**Bumble bee: Imidacloprid**	Treatment	1,16	39.59	< **0.001**
Binomial distribution	Year	2,16	1.32	0.390
	Site	4,16	1.01	0.436
	Treatment*Year	2,16	1.36	0.321
	Treatment*Site	4,16	1.40	0.392
**Bumble bee: Clothianidin**	Treatment	1,16	17.52	**0.007**
Binomial distribution	Year	2,16	6.29	0.073
	Site	4,16	2.36	0.152
	Treatment*Year	2,16	0.32	0.301
	Treatment*Site	4,16	1.21	0.380
**Bumble bee: Thiamethoxam**	Treatment	1,16	3.12	0.119
Binomial distribution	Year	2,16	0.46	0.774
	Site	4,16	1.27	0.295
	Treatment*Year	2,16	0.74	0.605
	Treatment*Site	4,16	1.16	0.342

Table S3: All collected and identified species of pollinators in watermelon fields from 2018-2020. Yearly columns from both the conventional management (CM) and integrated pest management (IPM) systems were summed in the total column. Species order was based on the total frequency observed. All pollinators were identified to the lowest taxonomic level, frequently at a species level except for several *Lasioglossum* species and hover flies (Syrphidae).

Species/morphospecies	2018		2019		2020		Total
	CM	IPM	CM	IPM	CM	IPM	
Apis mellifera	75	146	159	194	163	128	1381
Melissodes bimaculatus	66	156	93	180	48	133	997
Lasioglossum pilosum	4	63	27	73	59	84	469
Augochlora pura	49	232	3	24	4	46	389
Lasioglossum verstatum	12	24	27	52	26	92	338
Bombus impatiens	13	56	19	68	5	40	293
Lasioglossum imitatum	0	58	14	48	11	35	239
Syrphidae *spp.*	19	25	2	23	23	25	165
Lasioglossum brunei	9	31	2	27	18	15	149
Peponapis pruinosa	0	0	0	9	1	56	76
Halictus ligatus	2	1	3	20	2	11	64
Lasioglossum callidum	2	19	0	4	4	18	55
Halictus confusus	0	14	0	9	1	1	35
Lasioglossum luecocomum	1	4	0	6	0	17	34
Augochlorella aurata	0	0	0	8	1	11	29
Lasioglossum sp. 1	0	7	1	5	0	8	27
Halictus rubicundus	0	6	0	6	1	2	22
Lasioglossum oceanicum	0	0	0	5	0	8	18
Agapostemon splendens	1	3	0	3	0	6	16
Lasioglossum illioense	0	3	0	4	0	2	13
Triepeoious remigatus	0	1	1	2	2	1	12
Chaulioganthus pensylvanicus	2	2	0	2	1	1	11
Lasioglossum zephyrum	0	4	0	2	0	3	11
Halictus parallelus	0	1	0	1	1	4	9
Lasioglossum sp. 2	0	1	0	1	1	4	9
Calliopsis andyreniformis	0	3	0	0	0	4	7
Xylocopa virginica	0	1	0	2	0	1	6
Lasioglossum albipenne	0	2	0	0	0	4	6
Difourea marginata	0	2	0	1	0	0	4
Halictus sp.1	0	0	0	2	0	0	4
Lasioglossum sp. 3	0	1	0	0	0	3	4
Megachile brevis	1	1	0	0	1	0	4
Agapostemon virescens	0	1	0	1	0	0	3
Agapostemon sericeus	0	2	0	0	0	0	2

Holcopasite calliopsidis	0	1	0	0	0	1	2
Agapostemon texanus	0	0	0	0	0	1	1
Andrena asteris	0	0	0	0	0	1	1
Augochloropsis metalica	0	0	0	0	0	1	1
Ceratina calcarata	0	1	0	0	0	0	1
Hylaeus sp.	0	1	0	0	0	0	1
Nomada tyrrellensis	0	1	0	0	0	0	1

Table S4: List of non-neonicotinoid pesticides applied to the watermelon field during the experiment. Quantification procedures were specifically tailored to quantify neonicotinoid residues, resulting in an inability to detect the fungicide active ingredient chlorothalonil, or quantify non-neonicotinoid products. Percent detection of all honey bee wax and bumble bee nest material is reported from both conventional management (CM) and integrated pest management (IPM) systems.

	**Percent detection in honey bee wax** (n = 30)		**Percent detection in bumble bee nest material** (n = 30)	
**Product**	**CM**	**IPM**	**CM**	**IPM**
**Chlorothalonil**	na	na	na	na
**Fluopyram**	20%	33%	37%	23%
**Pyraclostrobin**	50%	30%	30%	43%
**Difenoconazole**	20%	3%	23%	27%
**Cyprodinil**	0%	0%	0%	0%
**Lambda cyhalothrin**	23%	0%	5%	0%

Table S5: Test statistics of all post hoc tests completed from model outputs of experimental response variables (Table S2). Listed statistics are for all Fishers LSD post-hoc tests run on all explanatory variables that were significantly different based on a level of $P < 0.05$ for all honey bee colonies (A), bumble bee colonies (B), wild pollinator surveys (C), and neonicotinoid residues (D). Any response variables without any significant differences detected were not further tested and not listed.

A. Honey bee colony parameters

Response Variable	Explanatory Variable(s)	MSE	Difference	*P*	*95% CI Lower*	*95% CI Upper*
**Final Weight Change**	Treatment					
	CM:IPM	21.31	-13.75	< **0.001**	-17.33	-10.18
	Year					
	2018:2019	21.31	-5.93	**0.011**	-10.31	-1.55
	2018:2020	21.31	-12.45	< **0.001**	-16.83	-8.08
	2019:2020	21.31	-6.53	**0.006**	-10.90	-2.15
	Site					
	NEPAC:PPAC	21.31	3.152	0.254	-2.498	8.803
	NEPAC:SEPAC	21.31	10.535	**0.001**	4.885	16.185
	NEPAC:SWPAC	21.31	5.865	**0.043**	0.215	11.515
	NEPAC:TPAC	21.31	3.363	0.225	-2.287	9.014
	PPAC:SEPAC	21.31	7.383	**0.014**	1.732	13.033
	PPAC:SWPAC	21.31	2.713	0.324	-2.938	8.363
	PPAC:TPAC	21.31	0.211	0.938	-5.439	5.861
	SEPAC:SWPAC	21.31	-4.670	0.099	-10.320	0.980
	SEPAC:TPAC	21.31	-7.172	**0.016**	-12.822	-1.521
	SWPAC:TPAC	21.31	-2.502	0.362	-8.152	3.149
**Average Brood Area**	Treatment					
	CM:IPM	3,397.32	-784.56	< **0.001**	-934.45	-634.66
	Year					
	2018:2019	3,397.32	-220.97	**0.021**	-404.56	-37.39
	2018:2020	3,397.32	-19.56	0.824	-203.14	164.03
	2019:2020	3,397.32	201.42	**0.033**	17.83	385.00
**At-Hive Mortality**	Treatment					
	CM:IPM	39.23	-11.79	**0.006**	-14.43	-3.13
	CM:PI	39.23	-6.99	**0.012**	-9.42	-2.91
	IPM:PI	39.23	-17.24	< **0.001**	-20.42	

B. Bumble bee colony parameters

Response Variable	Explanatory Variable(s)	MSE	Difference	*P*	*95% CI Lower*	*95% CI Upper*
**Colony Weight Change**	Treatment					
	CM:IPM	457.09	-108.52	< **0.001**	-125.07	-91.967
**Worker Weight**	Treatment					
	CM:IPM	375,927.02	-3,203.91	< **0.001**	-4,704.76	-1,703.06
	Year					
	2018:2019	375,927.02	-4,426.15	< **0.001**	-6,264.31	-2,587.99
	2018:2020	375,927.02	-1,558.93	**0.091**	-3,397.1	279.23
	2019:2020	375,927.02	2,867.21	**0.004**	1,029.05	4,705.37
**Queen Weight**	Treatment					
	CM:IPM	43,374.87	-335.191	< **0.001**	-496.405	-173.976
**Queen Count**	Treatment					
	CM:IPM	0.112	-0.483	**0.001**	-0.743	-0.224
**Live Worker Count**	Treatment					

	CM:IPM	326.48	-24.933	**0.002**	-38.920	-10.947
	Year					
	2018:2019	326.48	-19.975	**0.025**	-37.105	-2.845
	2018:2020	326.48	-8.875	0.288	-26.005	8.255
	2019:2020	326.48	11.100	0.188	-6.030	28.230
**Dead Worker Count**	Treatment					
	CM:IPM	722.7	30.183	**0.007**	9.374	50.993
**Worker Larvae Count**	Treatment					
	CM:IPM	143.71	-12.033	**0.014**	-21.313	-2.754
	Year					
	2018:2019	143.71	-4.250	0.440	-15.615	7.115
	2018:2020	143.71	-14.450	**0.016**	-25.815	-3.085
	2019:2020	143.71	-10.200	**0.075**	-21.565	1.165
**Egg Count**	Treatment					
	CM:IPM	993.52	-31.933	**0.014**	-56.333	-7.534
**Worker Honeypots**	Treatment					
	CM:IPM	1,492.64	-34.050	**0.028**	-63.956	-4.144
**Total Cells**	Treatment					
	CM:IPM	3,955.32	-79.400	**0.003**	-128.083	-30.717

C. Pollinator Survey

Response Variable	Explanatory Variable(s)	MSE	Difference	*P*	*95% CI Lower*	*95% CI Upper*
**Pollinator Abundance**	Treatment					
	CM:IPM	3.181	-4.392	**< 0.001**	-5.772	-3.011
	Site					
	NEPAC:PPAC	3.181	0.100	0.924	-2.083	2.283
	NEPAC:SEPAC	3.181	-5.053	**< 0.001**	-7.236	-2.871
	NEPAC:SWPAC	3.181	-1.043	0.326	-3.225	1.140
	NEPAC:TPAC	3.181	-1.046	0.325	-3.229	1.137
	PPAC:SEPAC	3.181	-5.153	**< 0.001**	-7.336	-2.971
	PPAC:SWPAC	3.181	-1.143	0.283	-3.326	1.040
	PPAC:TPAC	3.181	-1.146	0.282	-3.329	1.036
	SEPAC:SWPAC	3.181	4.011	**0.001**	1.828	6.193
	SEPAC:TPAC	3.181	4.007	**0.001**	1.824	6.190
	SWPAC:TPAC	3.181	-0.004	0.997	-2.186	2.179
**Species Richness**	Treatment					
	CM:IPM	9.41	-8.800	**< 0.001**	-11.174	-6.426
	Site					
	NEPAC:PPAC	9.41	-2.333	0.206	-6.087	1.421
	NEPAC:SEPAC	9.41	-5.167	**0.010**	-8.921	-1.413
	NEPAC:SWPAC	9.41	-6.667	**0.002**	-10.421	-2.913
	NEPAC:TPAC	9.41	-3.833	**0.046**	-7.587	-0.079
	PPAC:SEPAC	9.41	-2.833	0.129	-6.587	0.921
	PPAC:SWPAC	9.41	-4.333	**0.026**	-8.087	-0.579
	PPAC:TPAC	9.41	-1.500	0.409	-5.254	2.254
	SEPAC:SWPAC	9.41	-1.500	0.409	-5.254	2.254
	SEPAC:TPAC	9.41	1.333	0.462	-2.421	5.087
	SWPAC:TPAC	9.41	2.833	0.129	-0.921	6.587
**Shannon (H') Diversity**	Treatment					
	CM:IPM	0.079	-0.663	**< 0.001**	-0.881	-0.445
	Site					
	NEPAC:PPAC	0.079	-0.201	0.235	-0.546	0.144
	NEPAC:SEPAC	0.079	-0.353	**0.045**	-0.698	-0.008
	NEPAC:SWPAC	0.079	-0.768	**< 0.001**	-1.113	-0.423
	NEPAC:TPAC	0.079	-0.499	**0.007**	-0.844	-0.154

	PPAC:SEPAC	0.079	-0.152	0.363	-0.497	0.193
	PPAC:SWPAC	0.079	-0.567	**0.003**	-0.912	-0.222
	PPAC:TPAC	0.079	-0.299	0.085	-0.644	0.046
	SEPAC:SWPAC	0.079	-0.415	**0.021**	-0.760	-0.070
	SEPAC:TPAC	0.079	-0.146	0.382	-0.491	0.199
	SWPAC:TPAC	0.079	0.269	0.118	-0.076	0.614

D. Neonicotinoid residues

Response Variable	Explanatory Variable(s)	MSE	Difference	*P*	*95% CI Lower*	*95% CI Upper*
**Honey bee: Imidacloprid**	Treatment					
	CM:IPM	0.922	1.571	< **0.001**	0.828	2.314
**Honey bee: Clothianidin**	Treatment					
	CM:IPM	0.082	1.188	< **0.001**	0.966	1.410
**Honey bee: Thiamethoxam**	Treatment					
	CM:IPM	0.101	0.477	**0.001**	0.231	0.722
	Year					
	2018:2019	0.101	0.187	**0.007**	-0.114	0.488
	2018:2020	0.101	-0.022	0.877	-0.323	0.279
	2019:2020	0.101	0.209	0.160	-0.092	0.510
**Bumble bee: Imidacloprid**	Treatment					
	CM:IPM	0.048	0.442	< **0.001**	0.273	0.611
**Bumble bee: Clothianidin**	Treatment					
	CM:IPM	2.206	2.238	**0.001**	1.089	3.388

Figure S1: Map of Knox County IN using the USDA NASS Cropscape feature that illustrates land use for this county. Color key provides identification for two major row crops in the area along with watermelon, a key regional specialty crop.

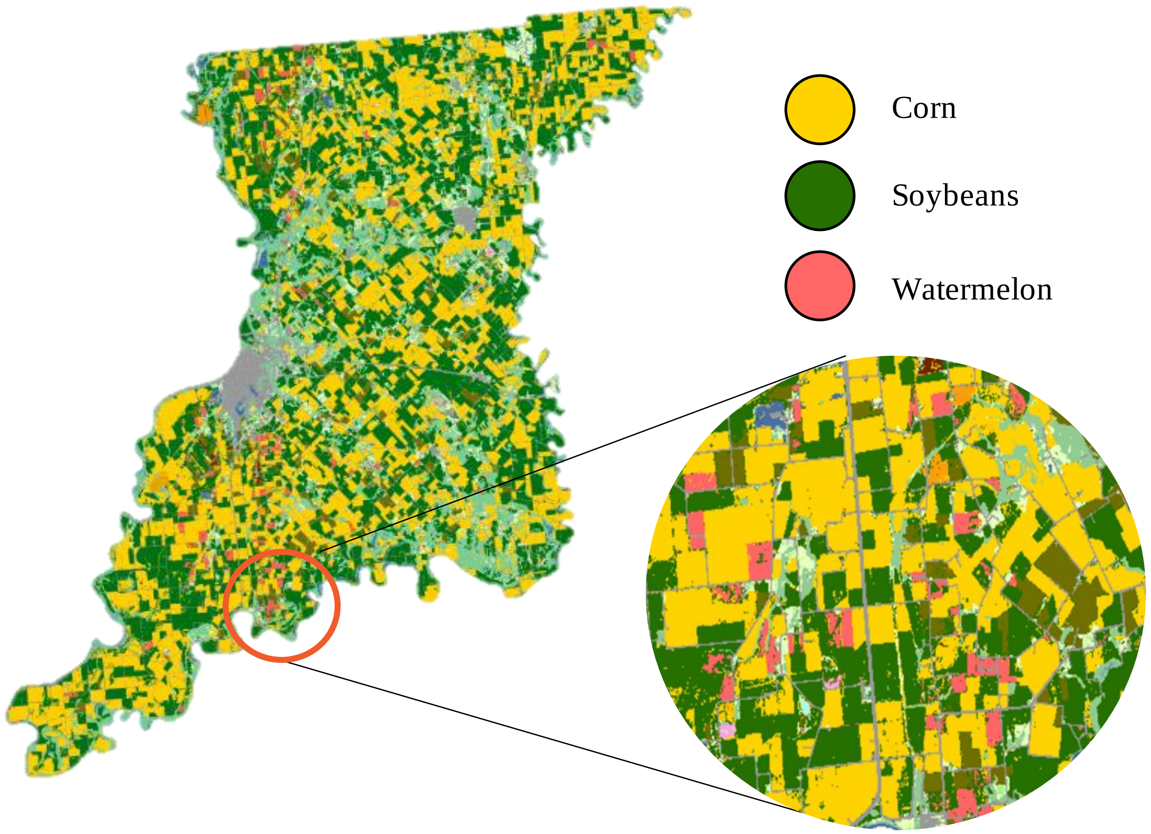

Figure S2: Pollinator communities with managed species separated to show abundance of managed colonies (A) and wild species (B) observed actively visiting watermelon flowers. To demonstrate the effect of managed species to community metrics managed species were removed from calculations of both species richness (C) and Shannon H' diversity (D). Each point within a cluster (n = 5) represents 5 weekly collections during that field season (75 total minutes). Whiskers within the plot show the average ± SEM of all sites within each cluster.

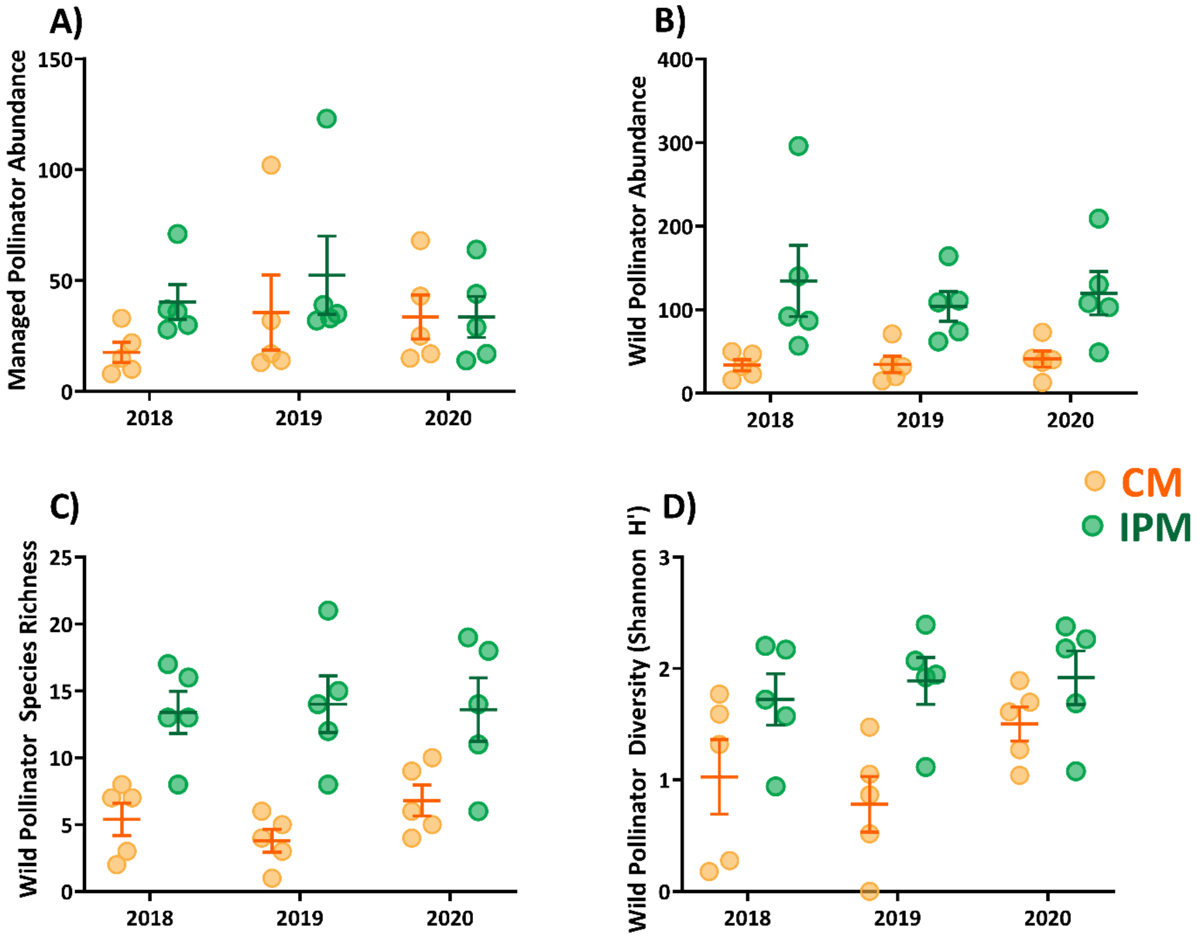

Supplement: Supplementary file 1 — Supplementary Information 1. [file 41598_2023_38053_MOESM1_ESM.pdf]
